# Methanolic Extract of Pien Tze Huang Induces Apoptosis Signaling in Human Osteosarcoma MG63 Cells via Multiple Pathways

**DOI:** 10.3390/molecules21030283

**Published:** 2016-03-01

**Authors:** Yong Fu, Li Zhang, Zhenqiang Hong, Haiyin Zheng, Nan Li, Hongjian Gao, Boyi Chen, Yi Zhao

**Affiliations:** 1 College of Osteopedics and Traumatology, Fujian University of Traditional Chinese Medicine, Fuzhou 350122, China; fyfjtcm@163.com (Y.F.); zzpzhhf@163.com (Z.H.); hmfjtcm@sina.com (N.L.); zzpzhhjm@163.com (H.G.); cbyfjtcm@sina.com (B.C.); zyfjtcm@sina.com (Y.Z.); 2 Integrative Medicine College, Fujian University of Traditional Chinese Medicine, Minhou Shangjie, Fuzhou 350122, China; zhyfjtcm@gmail.com

**Keywords:** osteosarcoma, Pien Tze Huang, PI3K, p-Akt, Bcl-2, Bax, caspase-3, caspase-9

## Abstract

Pien Tze Huang (PZH) is a well-known traditional Chinese formulation and has long been used as an alternative remedy for cancers in China and Southeast Asia. Recently, antitumor activity of PZH on several tumors have been increasingly reported, but its antitumor activity and the possible action mechanism on osteosarcoma remains unclear. After treatment with PZH, cell viability of MG-63 cells was dose-dependently inhibited compared to control cells. Moreover, a DNA ladder characteristic of apoptosis was observed in the cells treated with PZH, especially 500 μg/mL, 750 μg/mL. Further investigation showed that PZH treatments led to activation of caspase cascades and changes of apoptotic mediators Bcl2, Bax, and Bcl-xL expression. In addition, our results suggested that PZH activated PI3K/Akt signal pathway, and the phosphorylation of Akt and ERK1/2 were associated with the induction of apoptotic signaling. These results revealed that PZH possesses antitumoral activity on human osteosarcoma MG63 cells by manipulating apoptotic signaling and multiple pathways. It is suggested that PZH alone or combined with regular antitumor drugs may be beneficial as osteosarcoma treatments.

## 1. Introduction

Osteosarcoma is a common pediatric primary bone malignant tumor, whose incidence is the highest in primary bone malignant tumors [1]. It is most prevalent in children and young adults. Incidence rates for osteosarcoma in U.S. patients under 20 years of age are estimated at 5.0 per million per year in the general population [2]. The 5-year survival rate remains approximately 65%, and the rates after recurrence or metastasis are dramatically lowered to 30% [2,3]. Because of its high deterioration, poor prognosis and high damage, it becomes a difficult problem in medical field [4].

At present, there are mainly surgical operation and chemotherapy to treat this disease in clinic. Surgery, radiotherapy and chemotherapy in Osteosarcoma have made some progress, but these drugs are known to cause serious systemic toxicity, their limitations are more and more obvious [5,6]. There is still lack of effective therapy. Therefore, it is extremely urgent to develop favorable and low side-effects therapeutic agents. Traditional Chinese medicine have multi-ways, multi-targets, and fewer side effects in the treatment of this disease. Use of Pien Tze Huang (PZH) has gained increased interest as an alternative for treating Osteosarcoma.

PZH is a well-known traditional Chinese formulation first prescribed by a royal physician 450 years ago during the Ming Dynasty. PZH has properties of heat-clearing, detoxification, promotion of blood circulation and removal of blood stasis [7]. PZH has long been used as an alternative remedy for cancers in China and Southeast Asia and it has been listed as one of the national treasures in the catalogue of National Protected Traditional Chinese Medicines. Previously, our team has evaluated PZH on human osteosarcoma transplant mice model and osteosarcoma MG63 cell [8,9,10,11,12]. Our former research showed that it could promote cell differentiation, induce apoptosis of osteosarcoma *in vitro* study and inhibit osteosarcoma growth *in vivo* experiment. However, the mechanism of its anticancer activity, such as apoptosis, still remains largely unknown.

Herein, to further elucidate the mechanism of the tumoricidal activity of PZH, the current study was designed to confirm the potential effects of PZH and elucidate the underlying tumoricidal molecular mechanisms. Meanwhile, PZH is a complex combination of natural products, each of which contains numerous chemical compounds, so it is considered that the efficacy of PZH is associated with the synergistic or interactive work of numerous chemicals, including bile acids from calculus bovis, saponins from panax notoginseng, muscone from moschus and conjugated bile acids from snake gall [13,14,15,16,17,18]. Thus, the compounds of PZH was also determined as much as possible in this study.

## 2. Results

### 2.1. Chemical Characterization of PZH

**Figure 1:**
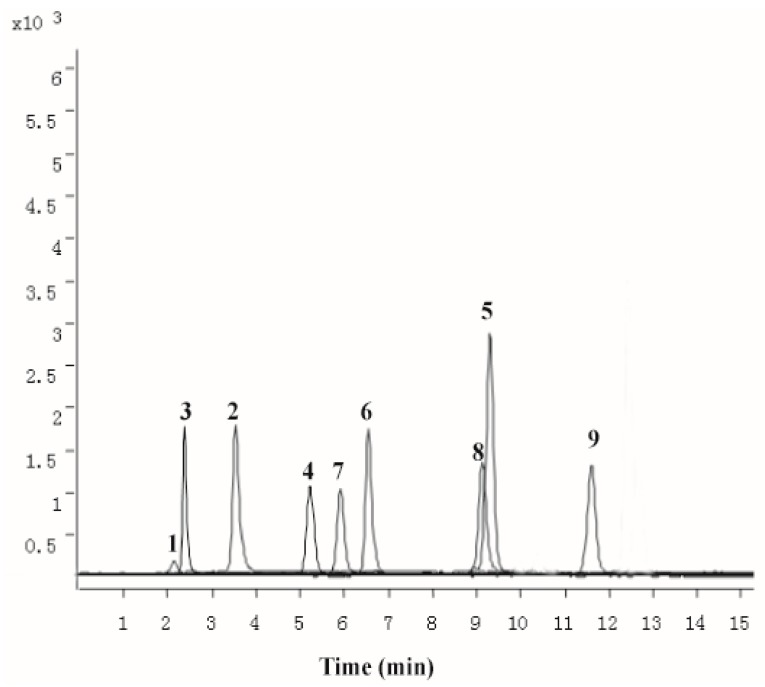
The multiple reaction monitoring (MRM) chromatograms of 10 standards of mixed standards. notoginsenoside R1(**1**); ginsenoside Rb1 (**2**); ginsenoside Rg1 (**3**); cholic acid (**4**); deoxycholic acid (**5**); hyodeoxycholic acid (**6**); ursodesoxycholic acid (**7**); chenodeoxycholic acid (**8**); muscone (**9**).

The methanolic extract obtained by ultrasonic extraction was chemically characterized by UPLC–QqQ-MS. LC–MS/MS MRM chromatogram of the 10 target compounds (notoginsenoside R1, ginsenoside Rb1, ginsenoside Rg1, ginsenoside Rg3, cholic acid, deoxycholic acid, hyodeoxycholic acid, ursodesoxycholic acid, chenodeoxycholic acid, sodium taurochenodeoxycholate, sodium tauroursodeoxycholate, muscone) was presented in Figure 1. The result revealed the average presence of notoginsenoside R1 (12.56 ± 0.19 mg/g), ginsenoside Rb1 (11.04 ± 0.28 mg/g), ginsenoside Rg1 (31.58 ± 1.23 mg/g), cholic acid (18.54 ± 0.31 mg/g), deoxycholic acid (1.55 ± 0.12 mg/g), hyodeoxycholic acid (0.298 ± 0.01 mg/g), ursodesoxycholic acid (0.158 ± 0.01 mg/g), chenodeoxycholic acid (3.29 ± 0.10 mg/g), sodium taurochenodeoxycholate (0.201 ± 0.09 mg/g), muscone (0.661 ± 0.04 mg/g) in PZH.

### 2.2. Morphology Observation

**Figure 2:**
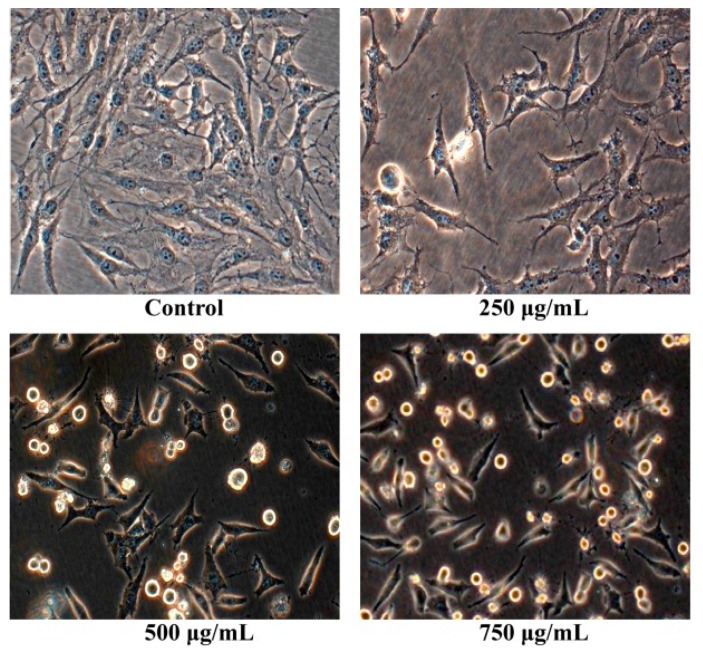
Pien Tze Huang (PZH) reduced cell viability of MG-63 cells. Cells viability treated with serial concentrations of PZH (250, 500 and 750 µg/mL) for 24 h was determined. Data are expressed as mean ± SD for three independent experiments.

After osteosarcoma MG63 cells were cultured with different concentrations of Pien Tze Huang solution (250 μg/mL, 500 μg/mL, 750 μg/mL) for 24 h, their shape were observed with the inverted phase contrast microscope. As shown in Figure 2, the human osteosarcoma MG63 cells without PZH exhibited normal morphology and grew well. While the human osteosarcoma MG63 cells with PZH suppressed cells proliferation, cells atrophied, became round and ultimately died. Moreover, the effect is dose-dependent.

### 2.3. Effect of PZH on Cells Proliferation

**Figure 3:**
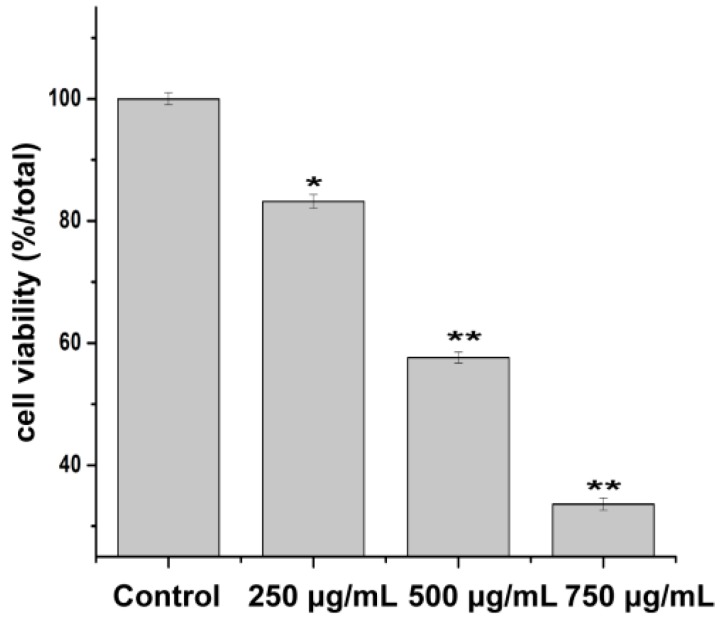
Morphology observation of MG63 cells. Cells treated with serial concentrations of PZH (250, 500 and 750 µg/mL) for 24 h were observed, * *p* < 0.05, ** *p* < 0.01 as compared with control.

Cell viability was evaluated by MTT assay. The results showed that the cell viability was inhibited by PZH in a concentration dependent manner (Figure 3). 500 μg/mL and 750 μg/mL of PZH significantly suppressed MG63 cells proliferation (*p* < 0.01) compared with the control group.

### 2.4. Effect of PZH on Cells Apoptosis

**Figure 4:**
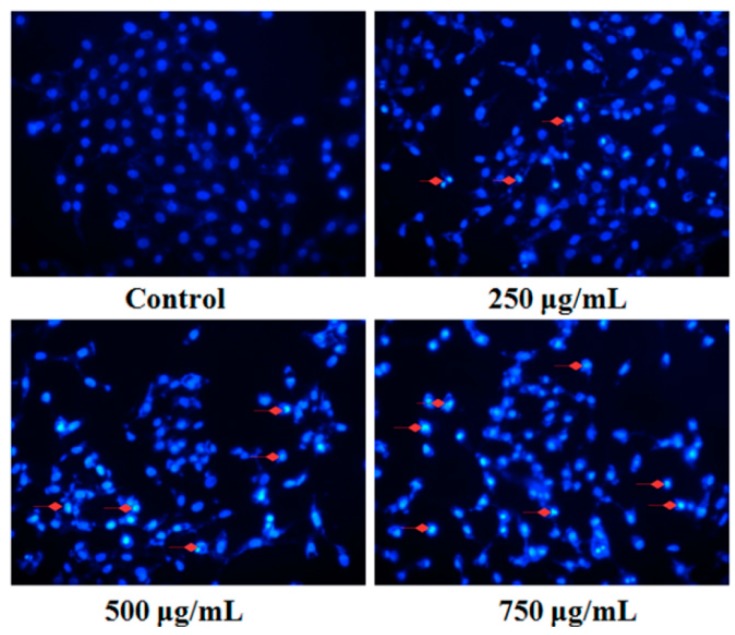
Fluorescent staining of MG63 cells with DAPI. Cells treated with serial concentrations of PZH (250, 500 and 750 µg/mL) for 24 h were stained with DAPI solution. Apoptotic body was labeled with red arrows.

Fluorescent staining and DNA fragmentation analysis were employed to evaluate the effect of PZH on cells apoptosis. As shown in Figure 4, morphology of MG63 cells in control group was normal, nuclei was round or oval and nuclear fragmentation phenomenon was not seen. However, the layer of PZH-treated cell became thinner with pyknotic nuclei, more nuclear fragmentation phenomenon was observed than in the control. Moreover, it was in a dose-dependent manner, especially in 750 μg/mL group, where there were a large number of apoptotic bodies (as shown in Figure 4 red arrows).

**Figure 5:**
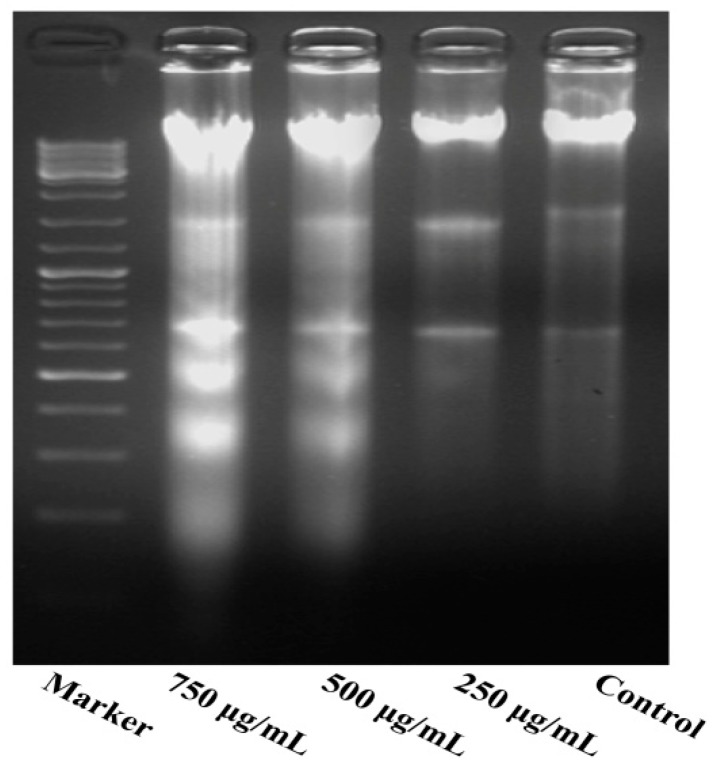
DNA fragmentation of MG63 cells. DNA of cells treated with serial concentrations of PZH (250, 500 and 750 µg/mL) for 24 h were analysed.

As shown in Figure 5, the results indicate a significant increase in inter-nucleosomal DNA fragmentation of MG63 cells. The DNA extracted from different concentrations of PZH-treated cells was subjected to agarose gel electrophoresis. No DNA fragmentation occurred in the control cells, while a DNA ladder characteristic of apoptosis was observed in the cells treated with PZH, especially 500 μg/mL, 750 μg/mL.

**Figure 6:**
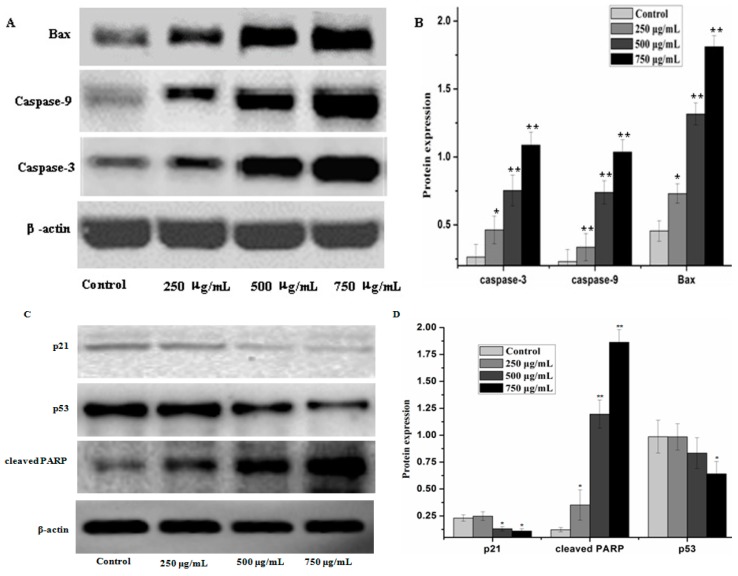
PZH-induced activation of caspase-3, caspase-9, p21, p53, cleaved-PARP and regulation pro-apoptotic protein Bax. The relative optical densities were indicated in B and D. Cells were treated with 250, 500 and 750 µg/mL PZH for 24 h and then were lyzed for the determination of the indicated protein levels by western blot. Actin was used as internal control. * *p* < 0.05, ** *p* < 0.01 *vs.* control group. Data are mean ± SD. (**A**) Protein expresion of Bax, caspase-3 and caspase-9; (**B**) The relative optical densities of Bax, caspase-3 and caspase-9; (**C**) Protein expresion of p21, p53 and cleaved-PARP; (**D**) The relative optical densities of p21, p53 and cleaved-PARP.

**Figure 7:**
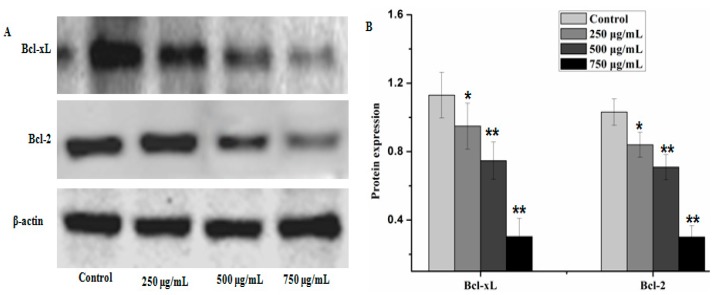
PZH inhibited anti-apoptotic protein Bcl-2 and Bcl-xL. The relative optical densities were indicated in B. Cells were treated with 250, 500 and 750 µg/mL PZH for 24 h and then were lyzed for the determination of the indicated protein levels by western blot. Actin was used as internal control. * *p* < 0.05, ** *p* < 0.01 *vs.* control group. Data are mean ± SD. (**A**) Protein expresion of Bcl-2 and Bcl-xL, (**B**) The relative optical densities of Bcl-2 and Bcl-xL.

Caspases and members of the family of the Bcl-2 play an important role in cells apoptosis. In the present study, caspase-3, caspase-9 and Bax which can potentially induce apoptosis in cells were analyzed by western blot analysis, as well as anti-apoptosis proteins, such as Bcl-xL and Bcl-2. The results showed that PZH elevated activity of caspase-3, caspase-9 and Bax in a dose dependent manner in MG63 cells. At 250 μg/mL, 500 μg/mL, 750 μg/mL of PZH, cleaved-PARP, caspase-3, caspase-9 and Bax activity increased significantly compared to the control (Figure 6). On the other hand, proteins expression of p21, p53, Bcl-xL and Bcl-2 were down-regulated by PZH (Figure 7).

### 2.5. Effect of PZH on Protein Expression of PI3K/Akt Signal Pathway

**Figure 8:**
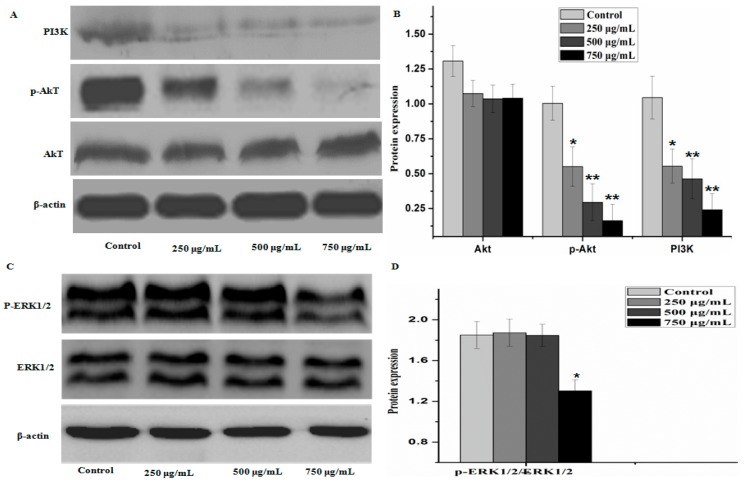
PZH triggered activation of PI3K/Akt signal pathway signaling. The relative optical densities were indicated in B and D. Cells were treated with 250, 500 and 750 µg/mL PZH for 24 h and then were lyzed for the determination of the indicated protein levels by western blot. Actin was used as internal control. * *p* < 0.05, ** *p* < 0.01 *vs.* control group. Data are mean ± SD. (**A**) Protein expresion of PI3K, p-AKT and AKT; (**B**) The relative optical densities of PI3K, p-AKT and AKT; (**C**) Protein expresion of ERK1/2 and p-ERK1/2; (**D**) The relative optical densities of ERK1/2 and p-ERK1/2.

To further elucidate the underlying mechanisms of activity of PZH, we determined its effect on the activation of PI3K/Akt signal pathway. As shown in Figure 8, there were no significant difference between the protein expression of Akt, ERK1/2 in control group and that of other groups, but the phosphorylation level of p-Akt and level of PI3K in MG63 cells were decreased as compared to control (*p* < 0.01), the phosphorylation level of p-ERK1/2 were obviously decreased at dose 750 μg/mL (*p* < 0.05). It was suggested that PZH exerts its activity probably via affecting PI3K/Akt signal pathway.

## 3. Discussion

In the physiological or pathological condition, the process of internal cells death occurs spontaneously and procedurally. This is called the apoptosis of cells [19]. The most important effectors of apoptosis are caspases, pro-apoptotic protein of Bcl family, which take part in a tightly regulated proteolytic cascade [20,21]. Previous studies have confirmed that caspases are the main executioners of apoptosis *in vivo*, and play a very important role in the process of apoptosis [22]. In this process, caspase-9 as the initiation factor of apoptosis, can activate the classic endogenous mitochondrial apoptosis pathway, then activate caspase-3 activity. Caspase-3 is the executive factor of apoptosis, they work together to induce the apoptosis of cells [23]. Therefore, cell apoptosis situation can be measured by detecting the expression of Caspase-3 and Caspase-9.

Bcl-2 family members are another main controllers of apoptosis in many cell types. They are divided into two groups: anti-apoptotic (Bcl-2, Bcl-xL *et al.*) and pro-apoptotic (Bax, Bad *et al.*) proteins [24]. The balance between pro-apoptotic and anti-apoptotic proteins is consider as to evaluate cell death or survival by controlling apoptosis [25]. It is proved that the activity of the Bcl-2 protein may be regulated through caspases cleavage under various circumstances [26]. In this study, osteosarcoma MG63 cells were cultured with different concentrations of PZH for 24 h. The detection of Western-blot analysis showed that activation of caspase-3, caspase-9 and Bax increased significantly by PZH and Bcl-2 and Bcl-xL proteins expression were inhibited significantly. It showed the conformity of the results with previous research [9,10,11,12]. Combined with the cells proliferation and DNA fragmentation analysis results, it is indicated that PZH could induce the apoptosis of osteosarcoma MG63 cells.

To evaluate the possible action mechanism of PZH, we investigated the proteins expression of P13K/Akt pathway and ERK1/2 by western blot. Much researches proved that there is a close relationship between P13K/Akt pathway, ERK1/2 and the proliferation, apoptosis, migration of tumor cell and the formation of tumor vessel [27,28,29]. Extracellular signal generates PIP3 (phosphatidylinositol 3,4,5-three phosphate) by P13K, PIP3 phosphorylate Akt. P-Akt can effectively control many related protein expression, cells proliferation, cells migration and invasion, inhibition of cell apoptosis [30]. Some researchers have found that p-Akt is high activated state in ovarian cancer, prostate cancer, colorectal cancer, hepatocellular carcinoma, follicular thyroid cancer and lung cancer [29,31]. As a downstream molecule of PI3K, Akt/PKB and ERK1/2 can promote the phosphorylation or inactivation of mitochondria and Bcl-2 family proteins, which play a role in apoptosis of tumor cells [32]. In this study, the proteins expression of PI3K and p-Akt revealed that PZH can inhibit the activation of PI3K and the phosphorylation of AKT. p53 and p21 function as tumor suppressor generally and are often up-regulated during the acute phase of apoptosis. However, p53 and p21 levels were reduced after PTH treatment. PTH possesses potent antitumoral activity, but p53 and p21 levels did not express in significant numbers. At present, we can not explain why, we should do further research in the future to explain this.

Taken together, these results and findings suggest that PZH possesses potent antitumoral activity on osteosarcoma MG63cells, attributing to induction of apoptosis and regulation of proteins expression of PI3K/Akt signal pathway.

## 4. Materials and Methods

### 4.1. Chemical Characterization of PZH

An ultrasonic extraction method was applied to PZH (Chinese FDA approval No: Z35020242, Zhangzhou Pian Tze Huang Pharmaceutical Co. Ltd., Zhangzhou, China), by mixing PZH powder (~0.050 g) with methanol (50 mL). Then extracted in an ultrasonic bath for 30 min. Additional methanol was added to make up the lost weight. Afterwards, the extracted solution was filtered and was centrifuged at 12,000 rpm for 10 min. The obtained supernatant was filtered through a 0.22 μm micropore membrane for analysis by UPLC–QqQ-MS [33].

### 4.2. Cell Culture and Treatment

The human osteosarcoma MG63 cells (the cell bank of Chinese Academy of Sciences, Shanghai, China) were cultured in RPMI 1640 medium supplemented with 10% (*v*/*v*) calf serum, 100 U/mL penicillin and streptomycin at 37 °C in humidified atmosphere of 95% air and 5% CO_2_. Briefly, cells were evaluated by counting trypan blue-excluding cells and then plated at a density of 2 × 10^4^ cells/well in 96-well trays and 5 × 10^5^ cells/well in 6-well trays, incubated at 37 °C for 24 h. The cultured cells were treated with or without PZH (250 µg/mL, 500 µg/mL, 750 µg/mL) for 24 h for the next experiments.

### 4.3. Cell Viability Assay

Conventional MTT reduction assay was used to determine cell viability. After treated, 10 µL MTT (0.5 mg/mL) was added to each culture well for additional 4 h incubation. Then the MTT was removed and the cells were dissolved with 100 µL of dimethylsulfoxide. Absorbance at 570 nm was measured in a microplate reader (Infinite M200 Pro, TECAN, Männedorf, Switzerland) after the formazan dye crystals were solubilized. Cell viability was expressed as a percentage of non-treated control. The assay was performed in triplicate and each group of eight wells in every assay.

### 4.4. Cells Apoptosis

#### 4.4.1. Fluorescent Staining

After treated, the culture-medium was removed, 4% paraformaldehyde solution was added to fix the cells at room temperature for 30 min. Then wash the cells three times with PBS. In the last wash, stain the cells with DAPI solution for 10 min. Finally, rinse the cells with PBS three times and observe the cells under the microscope using filters for DAPI. The experiment was performed in triplicate.

#### 4.4.2. DNA Fragmentation Analysis

After treated, the culture-medium was removed, then lysate and protease K sequentially were added. Cells were incubated at 55 °C in constant temperature water bath for 20 min. After that, RNA enzyme (10 μL) was added for reaction for 2 min. And phenol chloroform, isoamyl alcohol added for extraction. Finally, anhydrous ethanol was added to precipitate DNA. 5 μL DNA were analyzed using gel electrophoresis (2% agarose) for 3 h under voltage 20 V. And DNA bands were examined in a Gel Documentation System (BioRad, Model Gel Doc 2000, California, CA, USA). The experiment was performed in triplicate.

### 4.5. Western-Blot Analysis

Western blot analysis was used to evaluate protein level. Its method was similar to those described previously [34]. After treated, cells were collected and lysed by lysis buffer, then they were centrifuged at 12,000 *g* for 15 min. The supernatant was collected and the protein concentration was determined by the BCA method. Then protein mixed with loading buffer and incubated in 100 °C for 6 min. Ultimately, samples were stored in −20 °C for further analyses.

Cell lysates were analyzed for western blot analysis with primary antibodies (Caspase-3, Caspase-9, PI3K, Akt, p-Akt, p-Bad, Bcl-x, Bax, Bcl-2, p-21, p-53, cleaved PARP, ERK1/2 and actin). Finally they were evaluated using the ECL Western detection reagents. The protein expression was analyzed using Image Lab analysis software (Bio-Rad). The experiment was performed in six times.

### 4.6. Statistical Analysis

All data were presented as mean ± standard deviation and analyzed by SPSS16.0 statistical software (Chicago, IL, USA). One-way analysis of variance (ANOVA) were employed to determine significant differences between groups. A value of *p* < 0.05 was regarded as statistically significant.

## 5. Conclusions

In conclusion, to the best of our knowledge, the present study has proved that PZH is capable of inducing the apoptosis of osteosarcoma cell MG63 through multiple pathways.
